# Suicidality in clinically stable bipolar disorder and schizophrenia patients during the COVID-19 pandemic

**DOI:** 10.1038/s41398-022-02045-2

**Published:** 2022-07-29

**Authors:** Yu-Chen Li, Wei Bai, Hong Cai, Yuxuan Wu, Ling Zhang, Yan-Hong Ding, Juan-Juan Yang, Xiangdong Du, Zhen-Tao Zeng, Chang-Mou Lu, Ke-Xin Feng, Wen-Fang Mi, Lan Zhang, Huan-Zhong Liu, Lloyd Balbuena, Teris Cheung, Zhaohui Su, Feng-Rong An, Yu-Tao Xiang

**Affiliations:** 1 Department of Psychiatry, Xiamen Xianyue Hospital, Xiamen, China; 2 Unit of Psychiatry, Department of Public Health and Medicinal Administration, & Institute of Translational Medicine, Faculty of Health Sciences, University of Macau, Macao SAR, China; 3 Centre for Cognitive and Brain Sciences, University of Macau, Macao SAR, China; 4 Institute of Advanced Studies in Humanities and Social Sciences, University of Macau, Macao SAR, China; 5 Medical College of Soochow University, Suzhou, China; 6 Guangji Hospital Affiliated to Soochow University, Suzhou, Jiangsu province China; 7 Nanning Fifth People’s Hospital, Nanning, Guangxi province China; 8 Department of Psychiatry, Lanzhou University Second Hospital, Lanzhou, Gansu province China; 9 Department of Psychiatry, Chaohu Hospital, Anhui Medical University, Hefei, China; 10 School of Mental Health and Psychological Sciences, Anhui Medical University, Hefei, China; 11 School of Public Health, Lanzhou University, Lanzhou, Gansu province China; 12 Department of Psychiatry, University of Saskatchewan, Saskatoon, SK Canada; 13 School of Nursing, Hong Kong Polytechnic University, Hong Kong SAR, China; 14 School of Public Health, Southeast University, Nanjing, China; 15 The National Clinical Research Center for Mental Disorders & Beijing Key Laboratory of Mental Disorders, Beijing Anding Hospital & the Advanced Innovation Center for Human Brain Protection, Capital Medical University, Beijing, China

**Keywords:** Schizophrenia, Bipolar disorder

## Abstract

The coronavirus disease 2019 (COVID-19) pandemic has a disproportionate impact on vulnerable subpopulations, including those with severe mental illness (SMI). This study examined the one-year prevalence of suicidal ideation (SI), suicide plans (SP), and suicide attempts (SA) in bipolar disorder (BD) and schizophrenia (SCZ) patients during the pandemic. Prevalence rates were compared between the two disorders and associated factors were examined. A survey was conducted in six tertiary psychiatric hospitals and psychiatric units. People with a diagnosis of BD or SCZ were invited to participate. SI, SP, and SA (suicidality for short) were assessed and associated factors were examined using binary logistical regression. The 1-year prevalence of SI, SP and SA in BD patients were 58.3%, (95% CI: 54.1–62.6%), 38.4% (95% CI: 34.3–42.6%) and 38.6% (95% CI: 34.5–42.8%), respectively, which were higher than the corresponding figures in SCZ patients (SI: 33.2%, 95% CI: 28.6–37.8%; SP: 16.8%, 95% CI: 13.2–20.5%; SA: 19.4%, 95% CI: 15.5–23.3%). Patients with younger age, experience of cyberbullying, a history of SA among family or friends, a higher fatigue and physical pain score, inpatient status, and severe depressive symptoms were more likely to have suicidality. The COVID-19 pandemic was associated with increased risk of suicidality, particularly in BD patients. It is of importance to regularly screen suicidality in BD and SCZ patients during the pandemic even if they are clinically stable.

## Introduction

Suicide is a serious public health concern, and about 703,000 people worldwide died by suicide in 2019 [1]. It is one of the leading causes of death globally, with more than one in every 100 deaths (1.3%) caused by suicide [1]. Although China is one of the countries with the lowest suicide rates, China accounts for about one-sixth of suicide deaths worldwide [1]. Suicidality can be conceptualized as a continuum of thoughts and actions, from mild to severe, including suicidal ideation (SI), suicide planning (SP), suicide attempts (SA), and suicide deaths [2]. Non-fatal suicidality (i.e., SI, SP, and SA) is a strong predictor of subsequent suicide death [3]. A previous meta-analysis reported that the lifetime prevalence (95% CI) of SI and SA in China’s general population were 3.9% (2.5–6.0%) and 0.8% (0.7–0.9%), respectively [4]. Several factors are known to increase suicide risk including a previous SA, a family history of suicide and maltreatment, alcohol or drug abuse, and mental disorders [5–9].

Compared to those with no psychiatric disorders, individuals with severe mental illness (SMI) are more likely to commit suicide [10, 11]. In particular, both bipolar disorder (BD) and schizophrenia (SCZ) patients have higher rates of suicidality [11, 12]. Previous meta-analyses showed that the pooled lifetime and 1-year prevalence of SA in BD were 33.9% (95% CI: 31.3–36.6%), and 15.0% (95% CI: 8.2–21.8%), respectively [13]. In SCZ, the pooled lifetime prevalence of SI and SA were 34.5% (95% CI: 28.2−40.9%) and 26.8% (95% CI: 22.1–31.9%), respectively [14, 15]. Recent studies have suggested that the ongoing (COVID-19) pandemic might have increased suicidality in these susceptible populations [16, 17]. Measures intended to limit the spread of COVID-19, such as social distancing and lockdowns, may also decrease social support and increase loneliness [18], and increase maladaptive ways of coping [19]. Increased screen time, a higher consumption of junk food and alcohol, and tobacco smoking, may worsen the course of BD and SCZ [19, 20].

People with mental disorders have experienced disruptions in clinical care and medications as a result of travel restrictions and quarantine [21]. Moreover, the COVID-19 pandemic dramatically changed the landscape of care for patients with SMI. For instance, a survey involving seventeen regions of the world cautioned that the rise of telepsychiatry may limit psychiatric care opportunities for people without the technological means or aptitude [22]. COVID-19 has also led to overburdened healthcare professionals, compromising their mental health, which in turn results in suboptimal care for patients with mental disorders [23]. These factors may increase the risk of recurring affective episodes and/or suicidality in patients with BD or SCZ.

Financial challenges faced by patients brought about by COVID-19 may contribute to higher suicidality in BD and SCZ patients [24]. Recent studies found that a pre-existing SMI may increase the risk, severity, and mortality of COVID-19 infection [25]. Greater susceptibility to COVID-19 may worsen the stigma and isolation faced by BD and SCZ patients [26]—perhaps increasing suicidality. To the best of our knowledge, no study has examined suicidality in clinically stable BD and SCZ patients during the pandemic, although they account for majority of patients with these disorders.

Here, we examined and compared the 1-year prevalence of SI, SP, and SA in clinically stable BD and SCZ during the COVID-19 pandemic and identified factors associated with suicidality.

## Methods

### Participants and study site

This was a multicenter, cross-sectional study conducted in six tertiary psychiatric hospitals and psychiatric units in China between September 21, 2020 and October 8, 2021. In keeping with safety guidelines and following previous studies [27, 28], participants were assessed using the *QuestionnaireStar* program in the WeChat application. WeChat is a widely used social communication application in China and it was used for health monitoring during the pandemic. People entering hospitals are required to report their health status using a WeChat-generated code. We invited people visiting psychiatric units or admitted to psychiatric hospitals to participate in this study. Prospective participants scanned a Quick Response code (QR Code) that led to a study description and invitation. After providing electronic written informed consent, eligible patients were asked to complete a questionnaire. Participants were included if they were: (1) 18 years or above; (2) diagnosed with either BD or SCZ according to the International Classification of Diseases, Tenth Revision (ICD-10) [29]; (3) clinically stable as judged by their psychiatrists. Based on previous studies [30, 31], clinically stable patients were defined as those who had a less than 50% dose change in any main psychotropic medication in the last three months. These medications included antidepressants, mood stabilizers and antipsychotic medications. (4) able to read Chinese and understand the assessment questions. The study protocol received approval from the ethics committees of Beijing Anding Hospital and other participating hospitals.

### Measurements

Socio-demographic data, including age, gender, urban or rural residence, marital status, education, living arrangement (with family members/others or alone), employment status, health insurance status, self-reported health and economic status, family history of psychiatric disorders, cyberbullying, suicide attempts or death among family members or friends (henceforth, family-or-friend SA), were collected with a pre-designed data collection form. Clinical characteristics, such as inpatient or outpatient status, age of illness onset, fatigue, physical pain, depressive symptoms, quality of life and suicidality, were also collected.

Fatigue severity was measured with a numeric rating scale [32] with an answer ranging from “0” (no fatigue) to ‘10’ (extreme fatigue). Physical pain was assessed with a Visual Analogue Scale for Pain (VAS) [33] with anchors at “0” (no pain at all) and “10” (worst pain imaginable). The validated Chinese version of the Patient Health Questionnaire-2 (PHQ-2) [34, 35] was used to measure the severity of residual depressive symptoms, with a total score ranging from 0 to 6 and a higher score indicating more severity. Global quality of life (QOL) was measured with the first 2 items of the World Health Organization Quality of Life Scale Brief version (WHOQOL-BREF) [36, 37]. Higher scores reflect higher QOL.

Participants were asked about suicidality with standard questions adapted from the National Comorbidity Survey [38]. These questions were answerable with yes or no: (1) SI: “Over the past year, have you thought that you would be better off dead?” (2) SP: “Over the past year, have you made a plan for suicide?” (3) SA: “Over the past year, have you made an attempt for suicide?” These questions on suicidality have been widely used in other studies [39, 40]

### Statistical analysis

All analyses were performed using the R program [41]. Descriptive statistics (i.e., mean ± standard deviation (SD) for continuous variables and frequency counts (%) for categorical variables) summarized the participants’ socio-demographic and clinical characteristics. Continuous variables were examined for normality with the one-sample Kolmogorov-Smirnov test. Afterwards, univariate analyses (i.e., independent *t* tests, Mann–Whitney *U* tests, or Chi-square tests) were used to compare socio-demographic and disease-related variables between patients with and without suicidality within each diagnosis (i.e., BD/SCZ). We then examined if QOL differed between patients with and without suicidality by performing an analysis of covariance (ANCOVA), adjusting for variables in which the groups differed significantly in the univariate analyses. Within each diagnosis, binary logistic regression analysis was conducted to identify correlates of suicidality (SI, SP, and SA in separate models), adjusting for confounders. *P* < 0.05 (two-tailed) was set as the significance level.

## Results

Out of 949 patients invited, 905 (95.3%) satisfied the study criteria and completed the assessment. 513 patients had BD (mean age: 29.64 ± 11.58 years; male proportion: 20.5%) and 392 had SCZ (mean age: 32.42 ± 11.34 years; male proportion: 41.8%). The complete socio-demographic and clinical characteristics of the sample are summarized in Table 1.

**Table 1:** Socio-demographic and clinical characteristics of participants.

Variables	Bipolar disorder (*N* = 513)							Schizophrenia (*N* = 392)						
	Total	SI		SP		SA		Total	SI		SP		SA	
		No (*N* = 214)	Yes (*N* = 299)	No (*N* = 316)	Yes (*N* = 197)	No (*N* = 315)	Yes (*N* = 198)		No (*N* = 262)	Yes (*N* = 130)	No (*N* = 326)	Yes (*N* = 66)	No (*N* = 316)	Yes (*N* = 76)
	n (%)	n (%)	n (%)	n (%)	n (%)	n (%)	n (%)	n (%)	n (%)	n (%)	n (%)	n (%)	n (%)	n (%)
Male gender	105 (20.5)	46 (21.5)	59 (19.7)	73 (23.1)	32 (16.2)	71 (22.5)	34 (17.2)	164 (41.8)	117 (44.7)	47 (36.2)	133 (40.8)	31 (47.0)	134 (42.4)	30 (39.5)
Urban residents	404 (78.8)	167 (78.0)	237 (79.3)	251 (79.4)	153 (77.7)	250 (79.4)	154 (77.8)	225 (57.4)	157 (59.9)	68 (52.3)	191 (58.6)	34 (51.5)	180 (57.0)	45 (59.2)
Married	172 (33.5)	97 (45.3)	75 (25.1)^***^	126 (39.9)	46 (23.4)^***^	123 (39.0)	49 (24.7)^***^	137 (34.9)	98 (37.4)	39 (30.0)	123 (37.7)	14 (21.2)^*^	118 (37.3)	19 (25.0)^*^
College and above	323 (63.0)	134 (62.6)	189 (63.2)	203 (64.2)	120 (60.9)	199 (63.2)	124 (62.6)	151 (38.5)	106 (40.5)	45 (34.6)	129 (39.6)	22 (33.3)	126 (39.9)	25 (32.9)
Living with family members	417 (81.3)	185 (86.4)	232 (77.6)^*^	263 (83.2)	154 (78.2)	264 (83.8)	153 (77.3)	353 (90.1)	241 (92.0)	112 (86.2)	300 (92.0)	53 (80.3)^**^	287 (90.8)	66 (86.8)
Unemployed	311 (60.6)	114 (53.3)	197 (65.9)^**^	176 (55.7)	135 (68.5)^**^	173 (54.9)	138 (69.7)^***^	234 (59.7)	154 (58.8)	80 (61.5)	193 (59.2)	41 (62.1)	187 (59.2)	47 (61.8)
Health insurance	413 (80.5)	184 (86.0)	229 (76.6)^**^	262 (82.9)	151 (76.6)	262 (83.2)	151 (76.3)	321 (81.9)	215 (82.1)	106 (81.5)	273 (83.7)	48 (72.7)^*^	261 (82.6)	60 (78.9)
Inpatients	126 (24.6)	55 (25.7)	71 (23.7)	78 (24.7)	48 (24.4)	82 (26.0)	44 (22.2)	73 (18.6)	50 (19.1)	23 (17.7)	54 (16.6)	19 (28.8)^*^	58 (18.4)	15 (19.7)
Family history of psychiatric disorders	96 (18.7)	39 (18.2)	57 (19.1)	55 (17.4)	41 (20.8)	55 (17.5)	41 (20.7)	50 (12.8)	30 (11.5)	20 (15.4)	40 (12.3)	10 (15.2)	28 (12.0)	12 (15.8)
Cyberbullying	103 (20.1)	32 (15.0)	71 (23.7)^*^	43 (13.6)	60 (30.5)^***^	48 (15.2)	55 (27.8)^***^	95 (24.2)	50 (19.1)	45 (34.6)^***^	70 (21.5)	25 (37.9)^**^	79 (25.0)	16 (21.1)
Family members or friends’ SA	141 (27.5)	23 (10.7)	118 (39.5)^***^	48 (15.2)	93 (47.2)^***^	47 (14.9)	94 (47.5)^***^	51 (13.0)	21 (8.0)	30 (23.1)^***^	28 (8.6)	23 (34.8)^***^	28 (8.9)	23 (30.3)^***^
Family members or friends’ suicide	70 (13.6)	24 (11.2)	46 (15.4)	33 (10.4)	37 (18.8)^**^	37 (11.7)	33 (16.7)	32 (8.2)	16 (6.1)	16 (12.3) *	22 (6.7)	10 (15.2)^*^	22 (7.0)	10 (13.2)
Perceived health status														
Poor/Fair	375 (73.1)	138 (64.5)	237 (79.3)	216 (68.4)	159 (80.7)	214 (67.9)	161 (81.3)	286 (73.0)	181 (69.1)	105 (80.8)	233 (71.5)	53 (80.3)	224 (70.9)	62 (81.6)
Good	138 (26.9)	76 (35.5)	62 (20.7)^***^	100 (31.6)	38 (19.3)^***^	101 (32.1)	37 (18.7)^***^	106 (27.0)	81 (30.9)	25 (19.2)^***^	93 (28.5)	13 (19.7)^***^	92 (29.1)	14 (18.4)^***^
Perceived economic status														
Poor/Fair	455 (88.7)	185 (86.4)	270 (90.3)	275 (87.0)	180 (91.4)	275 (87.3)	180 (90.9)	371 (94.6)	248 (94.7)	123 (94.6)	309 (94.8)	62 (93.9)	300 (94.9)	71 (93.4)
Good	58 (11.3)	29 (13.6)	29 (9.7)	41 (13.0)	17 (8.6)	40 (12.7)	18 (9.1)^*^	21 (5.4)	14 (5.3)	7 (5.4)	17 (5.2)	4 (6.1)^*^	16 (5.1)	5 (6.6)
	Mean (SD)	Mean (SD)	Mean (SD)	Mean (SD)	Mean (SD)	Mean (SD)	Mean (SD)	Mean (SD)	Mean (SD)	Mean (SD)	Mean (SD)	Mean (SD)	Mean (SD)	Mean (SD)
Age (years)^#^	29.64 (11.58)	33.49 (11.77)	26.89 (10.64)^***^	32.02 (11.81)	25.83 (10.13)^***^	32.06 (11.73)	25.79 (10.24)^***^	32.42 (11.34)	33.65 (11.41)	29.93 (10.82)^***^	33.41 (11.59)	27.52 (8.48)^***^	33.31 (11.48)	28.70 (9.99)^***^
Age of onset (years)^#^	24.79 (10.16)	26.81 (25.00)	23.27 (9.14)^***^	26.60 (10.81)	21.89 (8.25)^***^	26.37 (10.91)	22.28 (8.26)^***^	25.66 (9.41)	26.29 (9.63)	24.37 (8.86)^*^	26.13 (9.55)	23.32 (8.42)^*^	26.31 (9.38)	22.93 (9.11)^***^
Fatigue^#^	4.78 (2.89)	3.50 (2.66)	5.69 (2.71)^***^	3.96 (2.71)	6.10 (2.69)^***^	3.88 (2.66)	6.21 (2.66)^***^	3.69 (2.55)	3.11 (2.32)	4.87 (2.61)^***^	3.39 (2.42)	5.18 (2.68)^***^	3.34 (2.40)	5.14 (2.67)^***^
Physical pain^#^	2.73 (2.61)	2.03 (2.39)	3.23 (2.66)^***^	2.21 (2.35)	3.57 (2.79)^***^	2.09 (2.25)	3.76 (2.82)^***^	2.10 (2.31)	1.64 (2.01)	3.02 (2.62)^***^	1.79 (2.11)	3.61 (2.72)^***^	1.81 (2.12)	3.29 (2.74)^***^
PHQ-2 total^#^	2.38 (2.06)	1.35 (1.70)	3.12 (1.97)^***^	1.64 (1.79)	3.56 (1.90)^***^	1.68 (1.81)	3.48 (1.93)^***^	1.74 (1.77)	1.26 (1.56)	2.71 (1.78)^***^	1.49 (1.63)	2.97 (1.92)^***^	1.47 (1.64)	2.87 (1.86)^***^
Global QOL^#^	5.50 (1.88)	6.26 (1.86)	4.95 (1.69)^***^	5.92 (1.84)	4.81 (1.74)^***^	5.90 (1.80)	4.86 (1.82)^***^	5.75 (1.75)	6.11 (1.71)	5.02 (1.61)^***^	5.93 (1.71)	4.86 (1.71)^***^	5.93 (1.71)	4.97 (1.74)^***^

*SI* suicidal ideation, *SP* suicide plan, *SA* suicide attempt, *M* mean, *SD* standard deviation, *PHQ-2* 2-item Patient Health Questionnaire, *QOL* quality of life.

Note: ^#^Mann–Whitney U test; ^*^P < 0.05; ^**^P < 0.01; ^***^P < 0.001.

### Patients with bipolar disorder

**Fig. 1:**
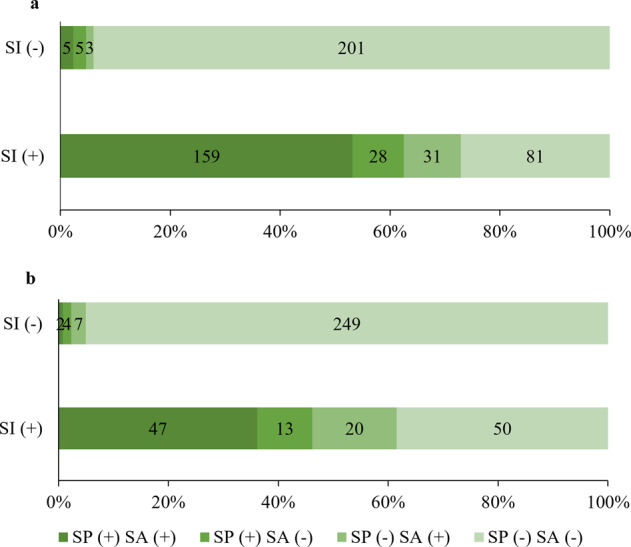
Distribution of suicide plan and suicide attempt in patients with and without suicidal ideation. Note: Numbers in bar graphs indicate patient counts. **a** shows counts of suicide plans (SP) and suicide attempts (SA) in BD patients by presence of SI. **b** shows SP and SA in SCZ by presence of SI. SI increases the risk for SP and SA in both disorders (*p* < 0.001).

Among BD patients, the 1-year prevalence (95% CI) of SI, SP and SA were: 58.3% (54.1%-62.6%), 38.4% (34.3%-42.6%), and 38.6% (34.5%-42.8%), respectively (Table 1). Univariate analyses revealed that BD patients having any form of suicidality were more likely to be unmarried, unemployed, report cyberbullying, have family-or-friend SA, report poor/fair health status, have younger age and age of onset, higher fatigue, higher physical pain, higher PHQ-2 and lower QOL scores (Table 1). Furthermore, patients without health insurance and those with family-or-friend SA were more likely to have SI or SP. Patients living with family members were less likely to have SI. BD patients with SI (compared to without) were more likely to have SP (χ^2^ = 176.579, *P* < 0.001) and SA (χ^2^ = 188.247, *P* < 0.001) (Fig. 1a).

**Fig. 2:**
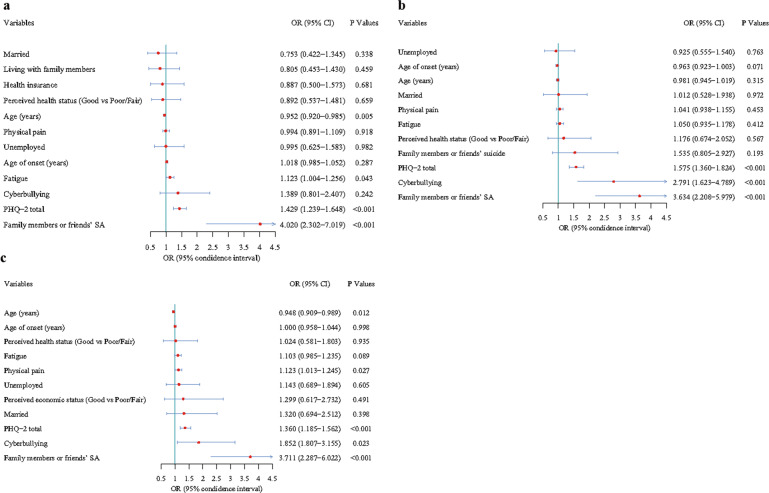
Independent correlates of suicidal ideation, suicide plan and suicide attempt in patients with bipolar disorder by multiple logistic regression analyses. Note: **a**, suicidal ideation; **b**, suicide plan; **c**, suicide attempt.

Three logistic regression models (one each for SI, SP, and SA) adjusted for confounders revealed that PHQ-2 total score and family-or-friend SA were positively associated with SI, SP and SA. Higher fatigue was associated with a higher risk of SI (OR = 1.123, 95%CI: 1.004–1.256, *P* = 0.043), while older age was inversely related to SI (OR = 0.952, 95%CI: 0.920–0.985, *P* = 0.005) and SA (OR = 0.948, 95%CI: 0.909–0.989, *P* = 0.012). Patients reporting cyberbullying had a higher risk of SP (OR = 2.791, 95%CI: 1.623–4.789, *P* < 0.001) and SA (OR = 1.852, 95%CI: 1.807–3.155, *P* = 0.023). Patients with higher physical pain score had a higher risk of SA (OR = 1.123, 95%CI: 1.013–1.245, *P* = 0.027). Please refer to Fig. 2a–c for multiple logistic regression models adjusted for various confounders.

ANCOVA revealed that BD patients with SI (compared to without) had lower QOL after controlling for confounders, (F _(1,513)_ = 8.179, *P* = 0.004).

### Patients with schizophrenia

In SCZ patients, the 1-year prevalence (95% CI) of SI, SP and SA were 33.2% (28.6–37.8%), 16.8% (13.2–20.5%), and 19.4% (15.5–23.3%), respectively (Table 1). Univariate analyses revealed that SCZ patients who were older, had a younger age of onset, had family-or-friend SA, reported poor/fair health status, had higher fatigue, higher physical pain, higher PHQ-2 total and lower QOL score were more likely to have SI, SP, and SA. Between SCZ patients with or without suicidality (i.e., any type of suicidality), there were significant differences in marital status, living status, health insurance status, patients' type, cyberbullying, family-or-friend suicide and self-reported economic status. SCZ patients with SI (compared to without) were more likely to have SP (χ^2^ = 119.394, *P* < 0.001) and SA (χ^2^ = 128.641, *P* < 0.001) (Fig. 1b).

**Fig. 3:**
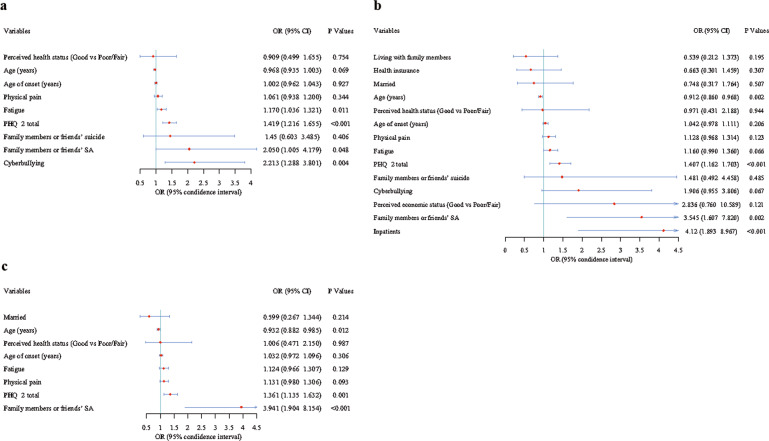
Independent correlates of suicidal ideation, suicide plan and suicide attempt in patients with schizophrenia by multiple logistic regression analyses. Note: **a**, suicidal ideation; **b**, suicide plan; **c**, suicide attempt.

Binary logistic regression analyses revealed that SCZ patients who had severe depressive symptoms and family-or-friend SA had a higher risk of SI, SP and SA. Older age was inversely associated with SP (OR = 0.912, 95%CI: 0.860-0.968, *P* = 0.002) and SA (OR = 0.932, 95%CI: 0.882–0.985, *P* = 0.012). A higher fatigue score (OR = 1.170, 95%CI: 1.036–1.321, *P* = 0.011) and cyberbullying (OR = 2.213, 95%CI: 1.288–3.801, *P* = 0.004) were associated with higher risk of SI. Inpatients had higher SP risk (OR = 4.12, 95%CI: 1.893–8.967, *P* < 0.001) compared to outpatients (Fig. 3).

Unlike BD, SCZ patients with SI, SP, and SA did not have significantly lower QOL when the ANCOVA controlled for confounding variables.

## Discussion

In this study, we examined the 1-year prevalence of SI, SP, and SA in BD and SCZ patients during the COVID-19 pandemic. The main findings are that (1) the prevalence of any type of suicidality in BD patients was significantly higher than that in SCZ patients, and (2) suicidality rates in these disorders were higher compared to pre-pandemic rates. The first finding was similar to the results reached by previous meta-analyses conducted before the COVID-19 pandemic [13, 15]. In these two studies [13, 15], patients with SCZ) had a lower one-year risk of SA (3.0%) compared to those with BD (15.0%). Moreover, we found that BD/SCZ patients with SI had a higher risk of SP and SA than those without SI, which is consistent with previous findings in BD/SCZ patients [42, 43].

Compared to previous meta-analyses, the one-year suicidality rates (SA: 38.6%, 95% CI: 34.5–42.8% in BD; SA SCZ: 19.4%, 95% CI: 15.5–23.3% in SCZ) in this study were higher than non-pandemic rates in BD (one-year SA: 15%, 95% CI: 8–22%) [13] and SCZ (one-year SA: 3.0%, 95% CI: 2.3–3.7%) [15]. Increased suicidality in these two disorders could be byproducts of regulations to curb the transmission of COVID-19. First, social distancing regulations and quarantine [18] had a particularly negative impact on vulnerable populations, who have typically smaller social support networks than the general population [44]. The above measures may have eroded social support resulting in higher suicidality. Second, BD and SCZ patients rely on health professionals for care and prescriptions. COVID-19 regulations may have hindered visits to hospitals and clinics [45] and increased suicidality is an indicator of lost care. Third, the pandemic has both triggered and increased fear of infection, anxiety, and depression in the general population [46] as well as in people with mental disorders [45]. People with mental disorders may be more susceptible to these negative emotions, which may in turn have increased the risk of suicidality. Fourth, stigma and discrimination towards mental illness may have been amplified during the COVID-19 pandemic [19, 47], leading to higher suicidality in the stigmatized group.

The finding that younger age is associated with higher suicidality in both disorders was previously observed in meta-analytic studies of BD and SCZ patients [48, 49]. Online communication became the dominant mode during the pandemic and especially among younger people. Although people with SMI can also benefit from internet communication [50], it brings risks such as hostile, derogatory comments and online harassment [51]. Furthermore, younger people have lower levels of mental resilience, which is crucial for blunting stress and promoting adaptation [52].

We found that cyberbullying increases SA, which is consistent with a previous study that found a positive association between cyberbullying and SI/suicide behaviour [53]. It has been suggested that positive mental health confers resilience to cyberbullying, thereby protecting against suicidality [53]. As opportunities to receive mental health and medications dwindled, younger people and targets of cyberbullying may have become less resilient. Like previous studies [48, 54], we found that a history of SA or deaths in family or friends increases the suicidality of BD and SCZ patients. Having such exposure probably brings trauma, increased rejection, shame, and stigma [55] thereby increasing their own suicidality risk.

The finding that higher fatigue increases risk of suicidality could be part of the higher stress and tension brought about by the pandemic [56]. Living with the pandemic for a year tested everyone’s resolve and many have felt depleted emotionally. Depressive symptoms are common in BD and SCZ patients, which are a well-known risk factor for suicidality in patients with SMI [48, 49]. Compared to non-psychiatric patients, patients with SMI had four times higher risk of reporting high COVID-19-related stress, and were 2–3 times more likely to have COVID-19-related anxiety and depressive symptoms [57]. Physical pain can also be related to depressive symptoms and stress because these conditions enhance pain sensitivity [58–60]. This combination of factors may have contributed to higher suicidality during the pandemic. The discordant findings between SCZ and BD regarding QOL and inpatient status as predictors of suicidality are difficult to explain since some important factors were not measured such as type and doses of psychotropic medications.

The main strength of this study is that we assessed suicidality in participants after one year of living with the pandemic in various areas in China. We also acknowledge several limitations. As a cross-sectional study, a causal effect cannot be inferred between suicidality and various factors. Also, we did not have information regarding patients’ access to medications and telepsychiatry. Differences in these factors may account for differences in suicidality. Finally, the limited range of psychiatric symptoms that we asked about could not be directly attributed to COVID-19 alone. This is in part due to the cross-sectional design of the survey, which was a necessity due to the unanticipated nature of the pandemic.

In conclusion, the pandemic has an outsized impact on SMI patients, resulting in a higher level of suicidality, especially in patients with BD. Several risk factors (e.g., younger age, inpatients, cyberbullying, and depressive symptoms) have been identified, and findings suggest that the importance of suicidality screening in patients with high risk and providing targeted effective interventions.
